# Sleep deprivation induces fragmented memory loss

**DOI:** 10.1101/lm.050757.119

**Published:** 2020-04

**Authors:** Jennifer E. Ashton, Marcus O. Harrington, Diane Langthorne, Hong-Viet V. Ngo, Scott A. Cairney

**Affiliations:** 1 Department of Psychology, University of York, Heslington, York YO10 5DD, United Kingdom; 2 Donders Institute for Brain, Cognition, and Behaviour, 6526 HR Nijmegen, The Netherlands; 3 York Biomedical Research Institute (YBRI), University of York, Heslington, York YO10 5DD, United Kingdom

## Abstract

Sleep deprivation increases rates of forgetting in episodic memory. Yet, whether an extended lack of sleep alters the qualitative nature of forgetting is unknown. We compared forgetting of episodic memories across intervals of overnight sleep, daytime wakefulness, and overnight sleep deprivation. Item-level forgetting was amplified across daytime wakefulness and overnight sleep deprivation, as compared to sleep. Importantly, however, overnight sleep deprivation led to a further deficit in associative memory that was not observed after daytime wakefulness. These findings suggest that sleep deprivation induces fragmentation among item memories and their associations, altering the qualitative nature of episodic forgetting.

Why are some memories remembered and others forgotten? Retroactive interference accounts of forgetting argue that learning and mental activity that occurs after encoding contributes to memory loss (Wixted 2004). Consistent with this view, rates of forgetting are typically reduced across sleep relative to wakefulness (Jenkins and Dallenbach 1924; Newman 1938; Barrett and Ekstrand 1972; Plihal and Born 1997; Gais et al. 2006; Tucker et al. 2006; Tamminen et al. 2010; Payne et al. 2012; Atherton et al. 2016; Cairney et al. 2018a,b), as sleep shelters new memories from competing information.

Given that forgetting is reduced by sleep, it is unsurprising that extended periods of sleep deprivation give rise to severe impairments in memory recall (Maquet et al. 2003; Gais et al. 2006; Tempesta et al. 2015, 2017; Harrington et al. 2018). In humans, empirical studies of sleep deprivation and memory often require participants to learn new information in the afternoon/evening, and then remain awake across the entire night (Maquet et al. 2003; Gais et al. 2006; Harrington et al. 2018). Hence, under these conditions, newly formed memories are subjected to a combination of retroactive interference and proactive interference (from events that occur prior to the encoding phase; Underwood 1957), leading to a substantial decline in recall accuracy.

To date, studies of sleep deprivation and memory have typically assessed forgetting for single items (e.g., images or words; Gais et al. 2006; Tempesta et al. 2015; Harrington et al. 2018). Episodic memory retrieval, in contrast, is critically dependent on the ability to recall associations between disparate features of prior experience (Tulving 1985). In recent work, pairwise event associations between locations, people, and objects were forgotten to a greater extent across daytime wakefulness than overnight sleep (Joensen et al. 2019). Yet, regardless of the postencoding delay (sleep or wake), forgetting invariably occurred in an all-or-none manner; when one element of an event (e.g., location) was remembered, the other elements of the same event (person and object) were also more frequently remembered than forgotten. Hence, although wakefulness increased overall rates of forgetting, it did not induce fragmentation among the memories that survived.

Sleep deprivation is known to amplify forgetting in episodic memory, but whether a protracted lack of sleep also leads to an irregular fragmentation of episodic representations has yet to be established. On account of the interference posed by waking activities occurring both before and after the critical learning episode (a deleterious combination of proactive and retroactive interference), sleep deprivation might open the door to fragmented forms of memory loss and, ultimately, alter the qualitative nature of forgetting.

Across two experiments, we investigated the impacts of sleep deprivation, as compared to sleep and routine daytime wakefulness, on memory for items and their associations. In Experiment 1, 27 healthy adults (10 male; mean ± SD age = 20.85 ± 3.29 yr) entered a within-subjects crossover design (sleep vs. wake, Fig. 1A). Conditions were separated by 1 wk and condition order was counterbalanced. Participants encoded adjective-object and adjective-scene pairs in the morning (08:00) or evening (20:00; Fig. 1B). The encoding phase included an immediate baseline test (T1), in which recognition memory (“old” or “new” judgments) for the adjectives was assessed. When an adjective was judged to be “old,” memory for the associated image category (object or scene) was also assessed. After T1, participants entered a 12 h delay of unsupervised daytime wakefulness (morning encoding) or overnight sleep at home (evening encoding). Participants were asked to refrain from caffeine and alcohol during this interval, and, if in the wake condition, refrain from napping. Adherence to these restrictions was confirmed via questionnaire. Participants in the sleep condition provided subjective estimations of hours slept (mean ± SD = 7.78 ± 0.90 h). Following the delay, participants were retested (T2). Whereas adjective forgetting between T1 and T2 reflects item memory loss, category forgetting reflects associative memory loss, or memory fragmentation, as memory for the base item persists.

**Figure 1.**
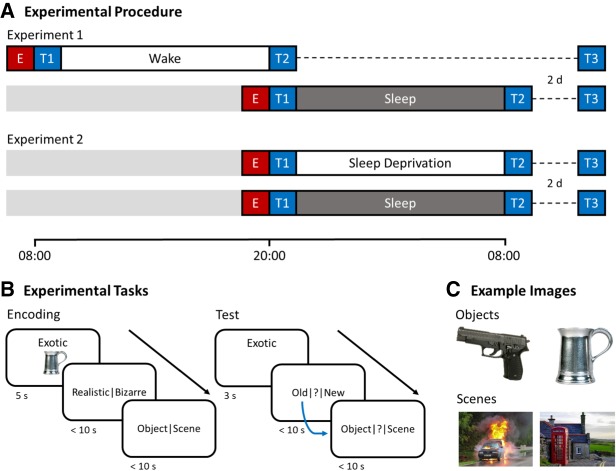
Experimental procedures, tasks, and example images. (*A*) The delay between test 1 (T1) and test 2 (T2) contained daytime wakefulness versus overnight sleep (Experiment 1) or overnight sleep deprivation versus overnight sleep (Experiment 2). A follow-up test (T3) occurred 2 d later. E = encoding. (*B*) One-hundred and twenty adjective-image pairs were presented at encoding. For each pair, participants were instructed to visualize the adjective and image interacting. They then reported whether the resultant mental image was realistic or bizarre, and indicated whether the presented image was an object or a scene. The same 120 adjectives from encoding were intermixed with 60 foil adjectives at retrieval. Participants first indicated if they recognized the adjective (“old”) or not (“new”), or were uncertain (“?”). For adjectives identified as “old,” participants also indicated whether the associated image was an object or scene (or “?”) and, if applicable, briefly described the image. (*C*) Example object and scene images (left side = negative; right side = neutral).

Experiment 2 (*n* = 28; 4 male; mean ± SD age = 19.43 ± 1.32 yr) followed identical procedures to Experiment 1, with the exception that retesting (T2) always took place in the morning following a night of sleep or total sleep deprivation. In both conditions, participants rose by 08:00 on the morning of the first session (∼12 h before encoding) and remained awake throughout the day (confirmed via wristwatch actigraphy). Resultantly, by T2 of the sleep deprivation condition, participants had been awake for ∼24 h. Across both experiments, we predicted that overnight sleep deprivation (vs. sleep and routine daytime wakefulness) would amplify adjective and category forgetting.

Sleep-deprived participants were monitored by a researcher throughout the overnight period. They were permitted to play games, watch movies and read. In the sleep condition, participants slept in a sleep laboratory and were monitored with polysomnography (Embla N7000; sampling rate = 200 Hz); permitting investigation of potential relationships between sleep stages and forgetting. Electrodes for electroencephalography (EEG) were attached at eight standardized locations: F3, F4, C3, C4, P3, P4, O1, and O2, each referenced to the contralateral mastoid (A1 or A2). Electrooculography (EOG) and electromyography (EMG) electrodes were also attached. Sleep data were segmented into 30 sec epochs and scored as wake, N1, N2, N3, or REM sleep in accordance with standardized criteria (see Supplemental_Table_S1.docx; Iber et al. 2007).

In both experiments, a follow-up test (T3) was administered 2 d after T2 (∼10:00) to assess item and associative memory loss following opportunities for recovery sleep. Participants completed the Stanford sleepiness scale (Hoddes et al. 1972) and a psychomotor vigilance test (Gagnepain et al. 2017) at each test phase (see Supplemental_Analysis_S1.docx and Supplemental_Table_S2.docx).

All behavioral tasks were implemented on a PC with MATLAB 2017a and Psychtoolbox 3.0.13 (Brainard 1997). At encoding, participants viewed 60 adjective-object pairs and 60 adjective-scene pairs in a randomized, intermixed order. Adjectives were selected from a database of 14,000 English lemmas (Warriner et al. 2013). Objects and scenes were selected from standardized image batteries (Lang et al. 2005; Marchewka et al. 2014) and online resources. Because previous work has suggested that negative affect can circumvent the impacts of sleep loss on item-level forgetting (Sterpenich et al. 2007; Vargas et al. 2019), we also investigated whether the effects of sleep deprivation on associative memory were modulated by emotion. The objects and scenes were therefore evenly subcategorized as negative or neutral. Assignment of images to negative and neutral subcategories was validated by an independent sample of healthy adults (*n* = 51, 4 male; mean ± SD age = 19.96 ± 5.29 yr). Emotional ratings (1 = highly negative, 5 = neutral, 9 = highly positive) were significantly lower for negative images (mean ± SEM = 2.98 ± 0.09) than neutral images (mean ± SEM = 5.61 ± 0.06; *t*_(50)_ = 26.00, *P* < 0.001, *d* = 3.64). All adjectives were emotionally neutral.

Each encoding trial began with a 1.5 sec fixation period. A randomly selected adjective was then displayed above a randomly selected object or scene image for 5 sec. Participants were instructed to visualize the adjective and image interacting, and then to indicate via keyboard press whether the mental image they generated was realistic or bizarre (to facilitate deep encoding; Craik and Lockhart 1972). To ensure that participants were able to differentiate between image categories, they were then asked to indicate whether the presented image was an object or scene. Image categorization performance was very high (both experiments: mean ± SEM = 96.83 ± 0.62%), and there were no differences in categorization accuracy between the sleep and wake conditions in Experiment 1 [*t*_(26)_ = 0.43, *P* = 0.67] or Experiment 2 [*t*_(27)_ = 1.14, *P* = 0.26]. Each adjective-image pair was presented once, and participants were required to make each of their responses within 10 sec.

A hierarchical approach was used at each test phase, permitting a distinction between item memory (adjectives) and associative memory (images associated with adjectives). T1 included 180 adjectives: 120 targets presented at encoding and 60 foils. Each trial began with a 1.5 sec fixation period, after which a randomly selected adjective was displayed for 3 sec. Participants were required to indicate whether the adjective was “old” (they recognized the adjective from encoding) or “new” (they did not recognize the adjective) within 10 sec. They were also able to indicate uncertainty by pressing “?”. This ensured that participants were reasonably confident in their “old”/“new” responses and discouraged guessing. Note that inclusion of the “uncertain” response at adjective recognition precluded calculation of the sensitivity index (*d*′) for item memory. Uncertainty data and analyses are available in Supplemental_Table_S3.docx and Supplemental_Analysis_S2.docx, respectively.

For each “old” response, participants indicated whether the image associated with that adjective at encoding was an object or scene, or pressed “?” if they were uncertain. After each “object” or “scene” response, participants provided a brief typed description of the image (e.g., “Pewter Mug” for Fig. 1C; see Supplemental_Analysis_S3.docx). For “new” or “uncertain” responses to adjectives, participants moved immediately to the next trial. The procedures for T2 and T3 were identical to those of T1, except that a new set of foil adjectives were used in each test.

Drawing on data from Experiment 1, we first investigated whether item memories were forgotten to a greater extent across a day of wakefulness relative to a night of sleep. To address this question, we isolated adjectives that were correctly recognized at the immediate test (T1) and then calculated the proportion of these adjectives that were forgotten (incorrect or “uncertain” responses) at the delayed test (T2). As expected, the resultant item loss proportion scores were higher after wakefulness than sleep [*t*_(26)_ = 2.44, *P* = 0.02, *d* = 0.47; Fig. 2A]. Behavioral data is displayed in Table 1.

**Figure 2.**
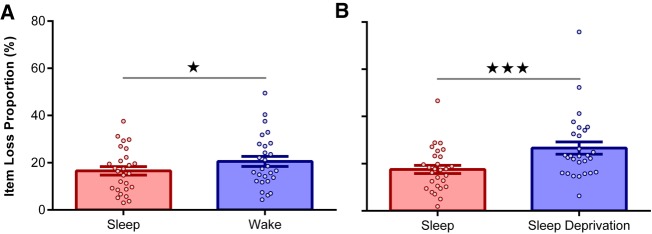
Item forgetting. (*A*) Experiment 1: Item forgetting was greater after a day of wakefulness relative to a night of sleep. (*B*) Experiment 2: Item forgetting was also greater after overnight sleep deprivation relative to sleep. Data points represent individual participants. Data are shown as mean ± SEM. (★) *P* < 0.05; (★★★) *P* < 0.001.

**Table 1.**
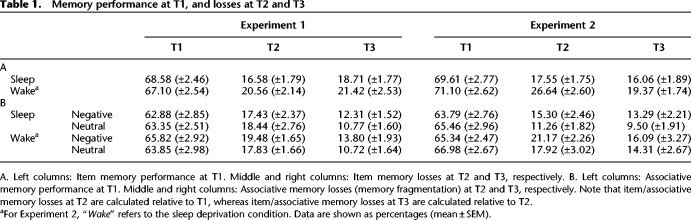
Memory performance at T1, and losses at T2 and T3

Turning to Experiment 2, we next examined whether overnight sleep deprivation also increased item forgetting relative to sleep. Indeed, when participants were deprived of sleep they exhibited a ∼50% proportional increase in item memory loss, as compared to when they slept [*t*_(27)_ = 4.58, *P* < 0.001, *d* = 0.87; Fig. 2B].

To assess whether overnight sleep deprivation was more conducive to item forgetting than routine daytime wakefulness, item loss proportion scores from both experiments were applied to a 2 (Delay: Sleep/Wake) × 2 (Experiment: One/Two) mixed ANOVA. A trend for the Delay*Experiment interaction suggested that the effects of wakefulness (vs. sleep) on item forgetting were amplified in Experiment 2 (overnight sleep deprivation) relative to Experiment 1 (daytime wakefulness; *F*_(1,53)_ = 3.93, *P* = 0.053, $\eta_{P}^{2}=0.07$). Unsurprisingly, the overall effect of wakefulness on item forgetting was highly significant [*F*_(1,53)_ = 25.72, *P* < 0.001, $\eta_{P}^{2}=0.33$], whereas general rates of item forgetting were comparable between experiments [*F*_(1,53)_ = 1.73, *P* = 0.19].

Next, we investigated whether sleep deprivation induced fragmentation among item memories and their associations. To probe this question, we first isolated adjectives that were correctly recognized at T1 and T2, and for which the associated image category (object or scene) was correctly retrieved at T1. We then calculated the proportion of these adjectives for which the image category was forgotten (incorrect or “uncertain” responses) at T2. The resultant fragmentation scores for Experiments 1 and 2 were submitted to separate 2 (Delay: Sleep/Wake) × 2 (Image Emotion: Negative/Neutral) repeated-measures ANOVAs.

In Experiment 1, fragmentation scores were comparable after daytime wakefulness and overnight sleep [*F*_(1,26)_ = 0.15, *P* = 0.71; Fig. 3A). Hence, although item forgetting was increased after a day of wakefulness (vs. overnight sleep), the waking delay had no impact on memory fragmentation. The fragmentation scores were unaffected by image emotion [Emotion main effect: *F*_(1,26)_ = 0.03, *P* = 0.86; Emotion*Delay interaction: *F*_(1,26)_ = 0.74, *P* = 0.40].

**Figure 3.**
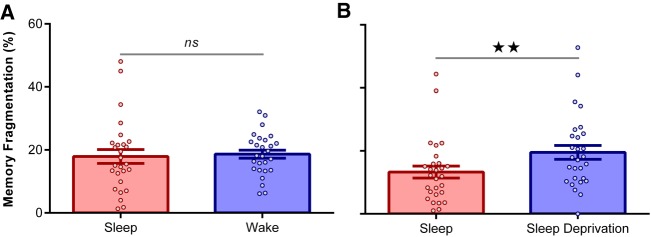
Memory fragmentation. (*A*) Experiment 1: Memory fragmentation was comparable after a day of wakefulness and a night of sleep. (*B*) Experiment 2: Memory fragmentation was greater after overnight sleep deprivation relative to sleep. Data points represent individual participants. Data are shown as mean ± SEM. (*ns*) not significant; (★★) *P* < 0.01.

Strikingly, however, fragmentation scores in Experiment 2 were significantly higher after sleep deprivation than sleep [*F*_(1,27)_ = 10.23, *P* = 0.004, $\eta_{P}^{2}=0.28$; Fig. 3B]. Thus, in contrast to routine daytime wakefulness (Experiment 1), overnight sleep deprivation appeared to induce fragmentation among item memories and their associations. Negative images were associated with greater fragmentation than neutral images [*F*_(1,27)_ = 4.45, *P* = 0.04, $\eta_{P}^{2}=0.14$], but this effect was not modulated by delay condition [*F*_(1,27)_ = 0.05, *P* = 0.83].

Consistent with the view that memory fragmentation was more prevalent after sleep deprivation than daytime wakefulness, a 2 (Delay: Sleep/Wake) × 2 (Experiment: One/Two) mixed ANOVA (collapsed across image emotion) revealed a significant Delay*Experiment interaction [*F*_(1,53)_ = 4.16, *P* = 0.05, $\eta_{P}^{2}=0.07$]. The overall effect of wakefulness on fragmentation scores was significant [*F*_(1,53)_ = 6.59, *P* = 0.01, $\eta_{P}^{2}=0.11$], whereas general rates of fragmentation were comparable between experiments [*F*_(1,53)_ = 0.62, *P* = 0.44]. Sleep duration (mean ± SEM) was 430.20 ± 6.45 min in the sleep condition of Experiment 2. There were no significant correlations between item or associative forgetting and time (min) spent in any stage of sleep [all *P* > 0.05].

In Experiment 2, alertness levels at T2 were reduced in the sleep deprivation (vs. sleep) condition, as indicated by the Stanford sleepiness scale and psychomotor vigilance test (see Supplemental_Analysis_S1.docx). The foregoing findings might thus be explained by between-condition differences in tiredness (and/or associated stress) on retrieval performance at T2. To address this possibility, we asked whether the effects of sleep deprivation on item forgetting and memory fragmentation observed at T2 were maintained 2 d later at T3 (when between-condition differences in tiredness were eliminated). Item loss proportion scores calculated between T1 and T3 (the proportion of correctly recognized adjectives at T1 that were forgotten at T3) were indeed higher in the sleep deprivation (vs. sleep) condition in Experiment 2 [*t*_(1,27)_ = 2.50, *P* = 0.02, *d* = 0.47]. Note that the same effect was observed when comparing the wake and sleep conditions in Experiment 1 [*t*_(26)_ = 2.06, *P* = 0.05, *d* = 0.40]. To compute fragmentation scores between T1 and T3 in Experiment 2, we first isolated adjectives that were correctly recognized at T1 and T3, and for which the associated image category was correctly retrieved at T1. We then calculated the proportion of these adjectives for which the image category was forgotten at T3. Importantly, fragmentation scores were higher in the sleep deprivation (vs. sleep) condition [*F*_(1,27)_ = 8.71, *P* = 0.01, $\eta_{P}^{2}=0.24$]. As before, a main effect of Emotion emerged [*F*_(1,27)_ = 5.47, *P* = 0.03, $\eta_{P}^{2}=0.17$], but there was no Emotion*Delay interaction [*F*_(1,27)_ = 1.60, *P* = 0.22]. Taken together, our findings suggest that the memory deficits associated with sleep deprivation were not simply due to excessive tiredness or stress at T2. It is nevertheless possible that high stress levels during consolidation contributed to a long-lasting fragmentation of memory.

We next investigated whether postlearning wakefulness or sleep deprivation (vs. sleep) led to any *further* impairment in item or associative memory 2 d later (i.e., impairments beyond those observed at T2). Item loss proportion scores calculated between T2 and T3 (the proportion of correctly recognized adjectives at T2 that were forgotten at T3) were comparable between the wake and sleep conditions in Experiment 1 [*t*_(26)_ = 1.22, *P* = 0.23]. However, sleep deprivation (vs. sleep) led to a trend toward additional item forgetting in Experiment 2 [*t*_(27)_ = 1.89, *P* = 0.07, *d* = 0.36]. Fragmentation scores calculated between T2 and T3 (the proportion of correctly retrieved image categories at T2 that were forgotten at T3, when the base adjective was correctly recognized at T2 and T3) were applied to a 2 (Delay: Sleep/Wake) × 2 (Emotion: Negative/Neutral) repeated-measures ANOVA. However, no significant effects emerged in Experiment 1 [Emotion: *F*_(1,26)_ = 2.17, *P* = 0.15; Delay: *F*_(1,26)_ = 0.25, *P* = 0.62; Emotion*Delay: *F*_(1,26)_ = 0.32, *P* = 0.58] or Experiment 2 [Emotion: *F*_(1,27)_ = 2.05, *P* = 0.16; Delay: *F*_(1,27)_ = 1.52, *P* = 0.23; Emotion*Delay: *F*_(1,27)_ = 0.36, *P* = 0.55].

Finally, we examined item and associative memory performance at T1 to ensure that the above effects were not driven by between-condition differences at baseline. Item memory performance was calculated as the proportion of “old” adjectives that were correctly identified as “old.” No differences were observed between the sleep and wake conditions in Experiment 1 [*t*_(26)_ = 0.79, *P* = 0.44] or Experiment 2 [*t*_(27)_ = 0.85, *P* = 0.41]. Associative memory performance was calculated as the proportion of correctly identified “old” adjectives for which the associated image category was also correctly retrieved. A 2 (Delay: Sleep/Wake) × 2 (Emotion: Negative/Neutral) repeated-measures ANOVA revealed no significant effects in Experiment 1 [Emotion: *F*_(1,26)_ = 0.32, *P* = 0.57; Delay: *F*_(1,26)_ = 0.59, *P* = 0.45; Emotion*Delay: *F*_(1,26)_ = 0.80, *P* = 0.38] or Experiment 2 [Emotion: *F*_(1,27)_ = 1.49 *P* = 0.23; Delay: *F*_(1,27)_ = 0.58, *P* = 0.45; Emotion*Delay: *F*_(1,27)_ < 0.001, *P* = 0.99].

Taken together, our findings suggest that sleep deprivation prompts a qualitative change in the nature of episodic forgetting. In Experiment 1, a routine day of wakefulness increased item-level forgetting relative to a night of sleep, but had no impact on associative memory when the base items survived. In Experiment 2, by contrast, overnight sleep deprivation (vs. sleep) not only increased item-level forgetting, but also increased associative memory loss when the base items remained unscathed. Hence, sleep deprivation appears to induce fragmentation among episodic representations that are typically forgotten in an all-or-none manner (Joensen et al. 2019).

Proactive and retroactive interference are thought to contribute to forgetting (Underwood 1957; Wixted 2004). Hence, a combination of these two sources of interference could have particularly deleterious effects on memory performance. In the sleep deprivation condition of Experiment 2, the encoding session was bookended by 12 h waking intervals (see Fig. 1A), providing scope for both proactive and retroactive interference. In the wake condition of Experiment 1, by contrast, encoding and retesting took place in the morning and following evening, respectively, meaning that the novel adjective-image associations were subjected only to retroactive interference. Across both experiments, sleep occurred soon after the evening encoding phase and seemingly ameliorated the impacts of proactive interference.

Wakeful experience is associated with a net increase in synaptic strength (De Vivo et al. 2017; Spano et al. 2019). A putative synaptic renormalization during sleep serves to globally downscale synaptic weights and, consequently, improve signal-to-noise ratios for synapses that were strongly potentiated as a result of prior learning (Tononi and Cirelli 2006). It has been suggested that this renormalization process constitutes an “efficient and smart” means of avoiding runaway potentiation and, importantly, separating meaningful information from unwanted interference (Tononi and Cirelli 2014). Amplified and fragmented forgetting following sleep deprivation could therefore be driven by excessive synaptic potentiation, which results from wakeful interference occurring before and after learning together with an absence of sleep-associated synaptic renormalization. Yet, it should be noted that time in N3—the sleep stage primarily implicated in synaptic renormalization (Tononi and Cirelli 2006, 2014)—was not correlated with item or associative memory performance in Experiment 2 of the current study.

Previous work has suggested that emotionally salient memories are more resistant to the effects of sleep deprivation than neutral memories (Sterpenich et al. 2007; Vargas et al. 2019). In the current study, by contrast, the impacts of sleep deprivation on memory fragmentation were comparable for negative and neutral images. This discrepancy may relate to the nature of the affective representation under scrutiny. Whereas previous studies have investigated the effects of sleep deprivation on central aspects of emotional memory (Sterpenich et al. 2007; Vargas et al. 2019), our findings relate to affective associations, which might be more susceptible to deterioration with sleep loss. Interestingly, memory fragmentation was generally greater for negative than neutral images in Experiment 2, which is consistent with earlier work (Bisby and Burgess 2013; Bisby et al. 2016), and the view that negative emotional content disrupts coherence among episodic representations (Bisby et al. 2018). Because the adjective stimuli used in this study were emotionally neutral, we could not determine how the emotional properties of item memories influence the susceptibility of their associations to sleep deprivation, although this is an interesting question for future research.

In conclusion, our findings suggest that sleep deprivation not only amplifies item-level forgetting, but induces fragmentation among item memories and their associations. Such fragmented memory loss might be due to a combination of proactive and retroactive interference, leading to severe and irregular impairments in episodic memory retrieval. More broadly, our findings offer novel insights into the cognitive impairments posed by insufficient sleep; an issue that is particularly pertinent when considering the global prevalence of chronic sleep deprivation (Bonnet and Arand 1995; Stranges et al. 2012; Liu et al. 2016), which is arguably at epidemic proportions.

## Data access

Study data are freely available via the following link: https://osf.io/s35f9/.

## Supplementary Material

Supplemental Material
