# Supplementary material for: Sleep deprivation induces fragmented memory loss
Source: Learn Mem. 2020 Apr;27(4):130–5. doi: 10.1101/lm.050757.119 (PMC7079571; doi:10.1101/lm.050757.119)
Supplement: Supplemental Material [file supp_27_4_130__index.html]

Sleep deprivation induces fragmented memory loss — Supplemental Material 

# Sleep deprivation induces fragmented memory loss

## Supplemental Material

- Supplemental\_Analysis\_S1\_R2.docx
- Supplemental\_Analysis\_S2\_R2.docx
- Supplemental\_Analysis\_S3\_R2.docx
- Supplemental\_Table\_S1\_R2.docx
- Supplemental\_Table\_S2\_R2.docx
- Supplemental\_Table\_S3\_R2.docx
