# Supplementary material for: Sleep deprivation induces fragmented memory loss
Source: Learn Mem. 2020 Apr;27(4):130–5. doi: 10.1101/lm.050757.119 (PMC7079571; doi:10.1101/lm.050757.119)
Supplement: Supplemental Material [file supp_27.4.130_Supplemental_Analysis_S1_R2.docx]

**Sleep Deprivation Induces Fragmented Memory Loss**

Supplemental Analysis S1

Participants completed the Stanford sleepiness scale (SSS; Hoddes et al. 1972) and a psychomotor vigilance test (PVT) immediately before each test (T1, T2 and T3). On each PVT trial, participants were presented with a fixation cross in the centre of the screen and two empty boxes on the left and right. After a variable delay (0.5-6.5 s), an arrow appeared in the centre of the screen and pointed left or right for 0.8 s. Following another variable delay (0.5-2 s), an asterisk appeared in one of the two boxes for 0.1 s and participants (upon seeing the asterisk) were instructed to press the spacebar as quickly as possible. The arrow pointed to the correct side of the screen (towards the box that the asterisk subsequently appeared in) on 75% of trials. There were 80 trials in total and task completion time was ~7 min.

Psychomotor vigilance was computed for each participant as the mean response time (RT) to congruent trials (trials in which the arrow pointed towards the correct side of the screen). PVT RTs and SSS scores were analysed using a 2 (Delay: Sleep/Wake) x 3 (Test: T1/T2/T3) repeated-measures ANOVA, separately for each experiment, when a significant interaction was observed, follow-up t-tests report a Holm-Bonferroni corrected p-value. Note that PVT data is missing for one participant in Experiment 1 due to a technical fault.

In Experiment 1, there was a significant main effect of Test on PVT RTs [*F*(2,50)=10.99, *p*<.001, *ƞ_p_^2^*=.31]. RTs at T1 were slower than those at T2 [*t*(25)=2.81, *p*=.01, *d*=.55] and T3 [*t*(25)=4.26, *p*<.001, *d*=.84]. RTs were also marginally slower at T2 compared to T3 [*t*(25)=2.05, *p*=.05, *d*=.40]. RTs did not differ significantly between the two delay conditions at any of the three tests [Delay main effect: *F*(1,25)=0.14, *p*=.71; Delay*Test interaction: *F*(2,50)=1.47, *p*=.24]. SSS scores in Experiment 1 were not influenced by Delay [*F*(1,26)=2.24, *p*=.15] or Test [*F*(2,52)=1.03, *p*=.36], and no interaction emerged [*F*(2.42,47.58)=1.32, *p*=.28, *Greenhouse-Geisser corrected*].

In Experiment 2, there was also a significant main effect of Test on PVT RTs [*F*(2,54)=23.67, *p*<.001, *ƞ_p_^2^*=0.47]. RTs at T1 were faster than those at T2 [*t*(27)=2.37, *p*=.025, *d*=0.45], but slower than those at T3 [*t*(27)=4.04, *p*<.001, *d*=0.76]. RTs at T2 were also slower than those at T3 [*t*(27)=7.44, *p*<.001, *d*=1.41]. There was no main effect of Delay [*F*(1,27)=2.36, *p*=.14], but a marginal Delay*Test interaction emerged [*F*(2,54)=2.91, *p*=.06, *ƞ_p_^2^*=.10]. At T2, RTs were slower in the sleep deprivation (vs sleep) condition [*t*(27)=2.35, *p*=.03, *d*=0.45]. However, RTs did not differ significantly between delay conditions at T1 [*t*(27)=0.45, *p*=.66] or T3 [*t*(27)=0.58, *p*=.57]. Regarding SSS scores in Experiment 2, main effects were observed for Delay [*F*(1,27)=68.27, *p*<.001, *ƞ_p_^2^*=0.72] and Test [*F*(2,54)=78.48, *p*<.001, *ƞ_p_^2^*=0.74]. These main effects were likely driven by a significant Delay*Test interaction [*F*(2,54)=88.56, *p*<.001, *ƞ_p_^2^*=0.77]. At T2, SSS scores were significantly higher in the sleep deprivation (vs sleep) condition [*t*(27)=14.13, *p*<.001, *d*=2.67]. However, SSS scores did not differ significantly between delay conditions at T1 [*t*(27)=1.49, *p*=.15] or T3 [*t*(27)=0.19, *p*=.85].

**References**

Hoddes, E., W. Dement, and V. Zarcone. 1972. The development and use of the Stanford sleepiness scale (SSS). Psychophysiology **9**:150.
