# Supplementary material for: Sleep deprivation induces fragmented memory loss
Source: Learn Mem. 2020 Apr;27(4):130–5. doi: 10.1101/lm.050757.119 (PMC7079571; doi:10.1101/lm.050757.119)
Supplement: Supplemental Material [file supp_27.4.130_Supplemental_Analysis_S2_R2.docx]

**Sleep Deprivation Induces Fragmented Memory Loss**

Supplemental Analysis S2

Across both experiments, participants provided an “uncertain” response on 12.24% of adjective recognition trials (SEM=1.43%; one sample *t*(54)=8.57, *p*<.001, *d*=1.16). Uncertain response proportions for adjectives in Experiments 1 and 2 were applied to separate 2 (Delay: Sleep/Wake) x 3 (Test: T1, T2, T3) repeated-measures ANOVAs, when a significant interaction was observed, follow-up t-tests report a Holm-Bonferroni corrected p-value. There was a significant main effect of Test in Experiment 1 [*F*(1.65,42.80)=16.55, *p*<.001, *ƞ_p_^2^*=0.39, *Greenhouse-Geisser corrected*] and Experiment 2 [*F*(2,54)=9.08, *p*<.001, *ƞ_p_^2^*=0.25]. Fewer uncertain responses were provided at T1, as compared to both T2 [Experiment 1: *t*(26)=3.89, *p*<.001, *d*=0.75; Experiment 2: *t*(27)=3.86, *p*<.001, *d*=0.73] and T3 [Experiment 1: *t*(26)=4.72, *p*<.001, *d*=0.91; Experiment 2: *t*(27)=2.82, *p*=.01, *d*=0.53]. Moreover, in Experiment 1, there were fewer uncertain responses at T2 relative to T3 [*t*(26)=2.57, *p*=.02, *d*=0.50]. There were no main effects of Delay [Experiment 1: *F*(1,26)=1.27, *p*=.27; Experiment 2: *F*(1,27)=1.38, *p*=.25]. The Delay*Test interaction was not significant in Experiment 1 [*F*(2,52)=0.55, *p*=.58], but a trend emerged in Experiment 2 [*F*(1.59,42.92)=3.04, *p*=.07, *Greenhouse-Geisser corrected*].

Across both experiments, participants provided an uncertain response on 35.62% of image-category retrieval trials (SEM=1.77%; one sample *t*(54)=20.12, *p*<.001, *d*=2.71). Uncertain response proportions for images in Experiments 1 and 2 were applied to separate 2 (Delay: Sleep/Wake) x 2 (Emotion: Negative/Neutral) x 3 (Test: T1, T2, T3) repeated-measures ANOVAs. There was a significant main effect of Test in Experiment 1 [*F*(1.24,32.28)=20.40, *p*<.001, *ƞ_p_^2^*=0.44, *Greenhouse-Geisser corrected*] and Experiment 2 [*F*(1.57,42.40)=25.22, *p*<.001, *ƞ_p_^2^*=0.48, *Greenhouse-Geisser corrected*]. Again, fewer uncertain responses were provided at T1 relative to T2 [Experiment 1: *t*(26)=5.92, *p*<.001, *d*=1.14; Experiment 2: *t*(27)=3.96, *p*<.001, *d*=0.75] and T3 [Experiment 1: *t*(26)=4.53, *p*<.001, *d*=0.87; Experiment 2: *t*(27)=5.92, *p*<.001, *d*=1.12]. In addition, for Experiment 2, there were fewer uncertain responses at T2 than T3 [*t*(27)=4.17, *p*<.001, *d*=0.79]. A significant Test*Emotion interaction emerged in Experiment 1 [*F*(2,52)=5.75, *p*=.01, *ƞ_p_^2^*=0.18]. There was an increase in uncertain responses between T2 and T3 for negative images [*t*(26)=3.27, *p*=.003, *d*=0.63], but not neutral images [*t*(26)=0.28, *p*=.78]. There were no main effects of Delay [Experiment 1: *F*(1,26)=0.10, *p*=.75; Experiment 2: *F*(1,27)=0.02, *p*=.89] or Emotion [Experiment 1: *F*(1,26)=0.09, *p*=.77; Experiment 2: *F*(1,27)=0.47, *p*=.50], and no other significant interactions [all *p*>.05].
