# Supplementary material for: Sleep deprivation induces fragmented memory loss
Source: Learn Mem. 2020 Apr;27(4):130–5. doi: 10.1101/lm.050757.119 (PMC7079571; doi:10.1101/lm.050757.119)
Supplement: Supplemental Material [file supp_27.4.130_Supplemental_Analysis_S3_R2.docx]

**Sleep Deprivation Induces Fragmented Memory Loss**

Supplemental Analysis S3

On the majority of trials, participants were able to accurately report the details of an image after making a correct object or scene response (Experiment 1 mean±SEM collapsed across conditions and test phases = 86.96±0.75%; Experiment 2 = 91.49±0.69%). There were no differences between the sleep and wake conditions at any test in Experiment 1 [T1: *t*(26)=0.72, *p*=.48, T2: *t*(26)=1.02, *p*=.32, T3: *t*(26)=0.24, *p*=.81] or Experiment 2 [T1: *t*(27)=1.70, *p*=.10, T2: *t*(27)=0.72, *p*=.48, T3: *t*(27)=1.02, *p*=.32]. Note that these analyses are collapsed across negative and neutral images.
