# Supplementary material for: Sleep deprivation induces fragmented memory loss
Source: Learn Mem. 2020 Apr;27(4):130–5. doi: 10.1101/lm.050757.119 (PMC7079571; doi:10.1101/lm.050757.119)
Supplement: Supplemental Material [file supp_27.4.130_Supplemental_Table_S1_R2.docx]

**Sleep Deprivation Induces Fragmented Memory Loss**

**Supplemental Table S1.** Sleep Stage Data.

| ***N1*** | ***N2*** | ***N3*** | ***REM*** | ***TST*** |
| --- | --- | --- | --- | --- |
| 27.73  (± 3.33) | 184.34  (± 5.94) | 115.46  (± 5.76) | 102.64  (± 4.25) | 430.18  (± 6.45) |

Abbreviations: N1, N2, N3 stages of non-REM sleep; REM, rapid eye movement sleep; TST, total sleep time. Data are shown in minutes (mean±SEM).
