# Supplementary material for: Sleep deprivation induces fragmented memory loss
Source: Learn Mem. 2020 Apr;27(4):130–5. doi: 10.1101/lm.050757.119 (PMC7079571; doi:10.1101/lm.050757.119)
Supplement: Supplemental Material [file supp_27.4.130_Supplemental_Table_S2_R2.docx]

**Sleep Deprivation Induces Fragmented Memory Loss**

**Supplemental Table S2.** Psychomotor vigilance and subjective sleepiness.

| **A** | ***Experiment 1*** | | |  | ***Experiment 2*** | | |
| --- | --- | --- | --- | --- | --- | --- | --- |
|  | *T1* | *T2* | *T3* |  | *T1* | *T2* | *T3* |
| *Sleep* | 319.00  (± 11.87) | 309.65  (± 11.96) | 307.34  (± 12.25) |  | 322.54  (± 8.24) | 323.29  (± 11.00) | 304.36  (± 8.77) |
| *Wake** | 321.56  (± 11.60) | 309.74  (± 13.63) | 298.37  (± 10.54) |  | 325.79  (± 7.13) | 343.82  (± 7.95) | 308.26  (± 8.41) |

| **B** | ***Experiment 1*** | | |  | ***Experiment 2*** | | |
| --- | --- | --- | --- | --- | --- | --- | --- |
|  | *T1* | *T2* | *T3* |  | *T1* | *T2* | *T3* |
| *Sleep* | 2.56  (± 0.23) | 2.44  (± 0.21) | 2.44  (± 0.16) |  | 2.57  (± 0.16) | 1.96  (± 0.17) | 1.82  (± 0.14) |
| *Wake** | 2.00  (± 0.13) | 2.41  (± 0.22) | 2.41  (± 0.24) |  | 2.29  (± 0.12) | 5.11  (± 0.16) | 1.86  (± 0.17) |

**A.** Psychomotor vigilance test response times (ms). **B.** Stanford sleepiness scale scores. Data are shown as mean±SEM. *For Experiment 2, “*Wake*” refers to the sleep deprivation condition.
