# Supplementary material for: Sleep deprivation induces fragmented memory loss
Source: Learn Mem. 2020 Apr;27(4):130–5. doi: 10.1101/lm.050757.119 (PMC7079571; doi:10.1101/lm.050757.119)
Supplement: Supplemental Material [file supp_27.4.130_Supplemental_Table_S3_R2.docx]

**Sleep Deprivation Induces Fragmented Memory Loss**

**Supplemental Table S3.** “Uncertain” Responses.

| **A** | ***Experiment 1*** | | |  | ***Experiment 2*** | | |
| --- | --- | --- | --- | --- | --- | --- | --- |
|  | *T1* | *T2* | *T3* |  | *T1* | *T2* | *T3* |
| *Sleep* | 7.16  (± 1.73) | 9.88  (± 2.13) | 10.80  (± 2.20) |  | 11.69  (± 1.91) | 14.41  (± 2.15) | 15.04  (± 2.22) |
| *Wake** | 8.21  (± 1.65) | 10.66  (± 1.90) | 12.94  (± 2.27) |  | 11.53  (± 1.85) | 18.41  (± 3.05) | 15.66  (± 2.90) |

| **B** | | ***Experiment 1*** | | |  | ***Experiment 2*** | | |
| --- | --- | --- | --- | --- | --- | --- | --- | --- |
|  |  | *T1* | *T2* | *T3* |  | *T1* | *T2* | *T3* |
| *Sleep* | *Negative* | 30.67  (± 2.91) | 36.68  (± 3.15) | 41.67  (± 3.64) |  | 32.51  (± 2.70) | 36.71  (± 3.07) | 39.65  (± 2.91) |
|  | *Neutral* | 30.89  (± 2.33) | 37.64  (± 3.26) | 37.38  (± 3.19) |  | 31.65  (± 2.99) | 34.64  (± 3.54) | 37.76  (± 3.32) |
| *Wake** | *Negative* | 27.82  (± 2.96) | 36.30  (± 2.70) | 39.33  (± 3.23) |  | 29.50  (± 2.20) | 37.04  (± 2.90) | 40.71  (± 3.42) |
|  | *Neutral* | 31.98  (± 2.85) | 38.70  (± 2.45) | 38.15  (± 3.10) |  | 30.03  (± 2.55) | 35.88  (± 3.41) | 41.50  (± 3.46) |

**A.** Proportion of adjective recognition trials for which participants provided an uncertain response. **B.** Proportion of image category trials for which participants provided an uncertain response. *For Experiment 2, “*Wake*” refers to the sleep deprivation condition. Data are shown as percentages (mean±SEM).
